# PHOTO DOCUMENTATION: USING NEW METHODS IN COLLABORATION WITH ART

**DOI:** 10.1093/geroni/igac059.974

**Published:** 2022-12-20

**Authors:** Melinda Heinz, Laura Gleissner, Nathan Benton

**Affiliations:** Upper Iowa University, Fayette, Iowa, United States; Upper Iowa University, Fayette, Iowa, United States; Upper Iowa University, Fayette, Iowa, United States

## Abstract

Narrative analysis has been used extensively in qualitative research, but photo analysis is relatively new. In the present study, older adults took photographs and wrote narratives for 15 days about something that brought meaning and purpose to their lives. This presentation explains how both methods were paired together and the challenges of seeking Institutional Review Board (IRB) when new and “unproven” approaches are used. In our case, IRB reviewers were particularly concerned with identifying information in photographs and recommended that personal information (e.g., medication bottle) should be blurred when sharing photographs. Discussion of how using diverse qualitative methods can create opportunities for new dissemination methods and partnerships with other disciplines will also be noted. In our study, several pop-up art exhibits of participant photos and narratives were hung in public community spaces to promote greater discussion and reflection on purpose and meaning in later life.

